# Supplementary material for: Cell-type-specific prediction of 3D chromatin organization enables high-throughput in silico genetic screening
Source: Nat Biotechnol. 2023 Jan 9;41(8):1140–50. doi: 10.1038/s41587-022-01612-8 (PMC10329734; doi:10.1038/s41587-022-01612-8)
Supplement: Supplementary file 3 — Cell-type-specific predictions. [file 41587_2022_1612_MOESM3_ESM.zip › Cell type-specific predictions/chr12_59000000.pdf]

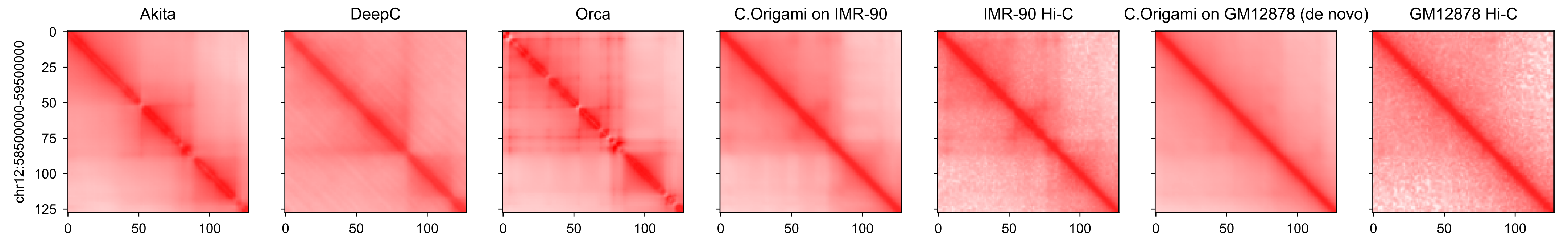

Akita
DeepC
Orca
C.Origami on IMR-90
IMR-90 Hi-C
C.Origami on GM12878 (de novo)
GM12878 Hi-C
chr12:58500000-59500000
0
25
50
75
100
125
0
50
100
